# Reconstructing an Ancestral Mammalian Immune Supercomplex from a Marsupial Major Histocompatibility Complex

**DOI:** 10.1371/journal.pbio.0040046

**Published:** 2006-01-31

**Authors:** Katherine Belov, Janine E Deakin, Anthony T Papenfuss, Michelle L Baker, Sandra D Melman, Hannah V Siddle, Nicolas Gouin, David L Goode, Tobias J Sargeant, Mark D Robinson, Matthew J Wakefield, Shaun Mahony, Joseph G. R Cross, Panayiotis V Benos, Paul B Samollow, Terence P Speed, Jennifer A. Marshall Graves, Robert D Miller

**Affiliations:** **1**Centre for Advanced Technologies in Animal Genetics and Reproduction, Faculty of Veterinary Science, The University of Sydney, Camden, Australia; **2**ARC Centre for Kangaroo Genomics, Research School of Biological Sciences, The Australian National University, Canberra, Australia; **3**The Walter and Eliza Hall Institute of Medical Research, Parkville, Australia; **4**Department of Biology, University of New Mexico, Albuquerque, New Mexico, United States of America; **5**Department of Genetics, Southwest Foundation for Biomedical Research, San Antonio, Texas, United States of America; **6**National Centre for Biomedical Engineering Science, National University of Ireland, Galway, Ireland; **7**Department of Human Genetics, Graduate School of Public Health, University of Pittsburgh, Pittsburgh, Pennsylvania, United States of America; **8**Department of Veterinary Integrative Biosciences, College of Veterinary Medicine and Biomedical Sciences, Texas A&M University, College Station, Texas, United States of America; Whitehead Institute for Biomedical ResearchUnited States of America

## Abstract

The first sequenced marsupial genome promises to reveal unparalleled insights into mammalian evolution. We have used theMonodelphis domestica (gray short-tailed opossum) sequence to construct the first map of a marsupial major histocompatibility complex (MHC). The MHC is the most gene-dense region of the mammalian genome and is critical to immunity and reproductive success. The marsupial MHC bridges the phylogenetic gap between the complex MHC of eutherian mammals and the minimal essential MHC of birds. Here we show that the opossum MHC is gene dense and complex, as in humans, but shares more organizational features with non-mammals. The Class I genes have amplified within the Class II region, resulting in a unique Class I/II region. We present a model of the organization of the MHC in ancestral mammals and its elaboration during mammalian evolution. The opossum genome, together with other extant genomes, reveals the existence of an ancestral “immune supercomplex” that contained genes of both types of natural killer receptors together with antigen processing genes and MHC genes.

## Abstract 2

Opossum genomic sequence data permit researchers to fill gaps in our knowledge of how the Major Histocompatibility Complex evolved within the mammalian lineage.

## Introduction

The major histocompatibility complex (MHC) is a multigene complex critical to vertebrate immunity. The MHC is the most gene-dense and polymorphic region of the mammalian genome and is associated with resistance to infectious diseases, autoimmunity, transplantation, and reproductive success [1]. Loci contained within the MHC have been historically grouped into three classes of genes called Class I, II, and III. The three classes of loci are distinguished based on both structure and function of their encoded proteins. Class I molecules can be divided into classical and non-classical molecules. Classical Class I (Class Ia) loci are ubiquitously expressed and encode receptors that typically bind and present endogenously synthesized peptides to antigen specific CD8^+^ cytotoxic T cells. Non-classical Class I (Class Ib) loci encode molecules that often perform functions other than antigen presentation. Class Ib loci may be located outside the MHC, and tend to be non-polymorphic and not ubiquitously expressed. Classical Class II genes encode receptors that present exogenously derived peptides to CD4^+^ helper T cells, whereas non-classical Class II genes participate in antigen presentation pathways. The MHC also contains several genes encoding molecules that participate in the processing of peptides for presentation to the immune system. The Class III genes encode a variety of immune and non-immune system–related molecules, most of which are not involved in antigen presentation, and include cytokines and components of the complement system. The three classes of loci are been used to define regions within the MHC, i.e. Class I, II, and III MHC regions.

Comparative analyses of the MHC organization across distantly related species has revealed lineage specific rearrangements within the region and changes in gene complexity. Detailed information on MHC organization is currently available for seven species of eutherian (“placental”) mammals, two birds, five teleost fish, and sharks [2–5]. There are major differences in organization and complexity between eutherian and non-mammalian MHC regions, and reconstructing the evolutionary history of this region has been difficult. The highly complex eutherian MHC is ordered along the chromosome as the Class I–III–II regions. The eutherian MHC is large and gene dense; for example, the human MHC contains 264 genes and pseudogenes over the 3.6 Mb region. In non-mammals, the MHC generally contains fewer genes than is found in mammals and the Class I and II regions are adjacent. Teleost fish are the exception. Their Class I and II regions are unlinked. Of the MHC regions completely sequenced, the chicken MHC is the least complex, containing only 19 genes over 92 kb [6].

In eutherians, the Class I region contains a set of framework genes whose presence and order are conserved among species; and between this framework, the Class I genes have expanded and diversified [7]. These Class I region framework genes have not been reported in the MHC of non-mammals. In eutherians, the Class II region contains the antigen processing genes*(TAP1, TAP2, PSMB8,* and*PSMB9),* which process endogenously synthesized peptides for presentation on Class I molecules. In non-mammals, however, the antigen processing genes are found in the Class I region, and their proximity is thought to have influenced Class I gene evolution [8].

Analysis of MHC structure in mammals distantly related to eutherians (marsupials and monotremes) would bridge a 200 million y gap between eutherians and non-mammalian vertebrates [9], and lead to a new understanding of the evolutionary forces that shaped the complex eutherian MHC. The availability of the opossum genome sequence provides the first opportunity to bridge this phylogenetic gap and provide insight into the evolutionary history of the mammalian MHC. Here we report that the opossum MHC region is similar to the eutherian MHC in both size and gene complexity; however, it also contains organizational features more like those found in non-mammals, revealing a likely ancestral organization in mammals. This analysis is the deepest comparison of the MHC region within mammals undertaken to date.

## Results/Discussion

The 3.6 Mb MHC region on human Chromosome 6 contains 140 genes between flanking markers*MOG* and*COL11A2* [10]. We found that the opossum MHC region bounded by the same flanking markers spans 3.95 Mb and contains 114 genes, recognized by homology to known genes from other species and/or the presence of open reading frames (ORF). Eighty-seven of these genes are shared with human MHC (Figure 1). A list of putative opossum MHC gene transcripts and our opossum MHC genome browser containing annotation are located athttp://bioinf.wehi.edu.au/opossum. The opossum MHC is located on Chromosome 2q near the centromere, oriented with*MOG* proximal (Figure 2) [11]. Physical mapping of 19 bacterial artificial chromosome (BAC) clones, corresponding to loci spaced along the entire scaffold, confirmed the accuracy of the assembly.

**Figure 1:**
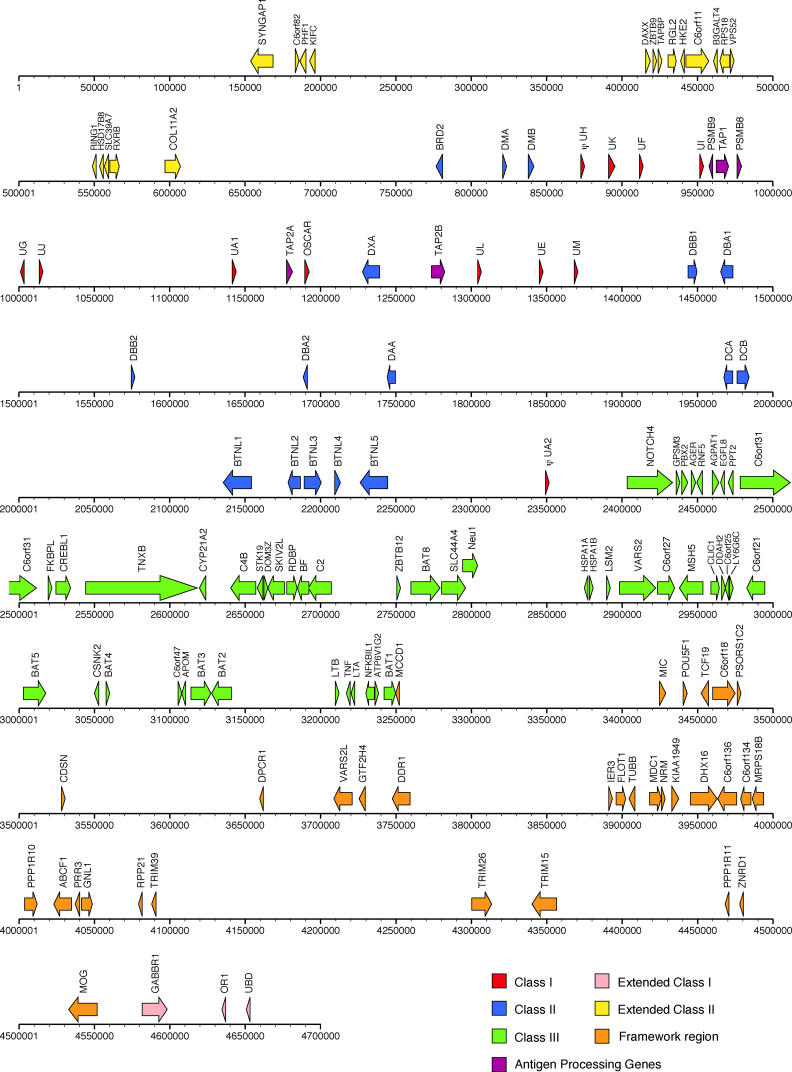
Map of the Opossum MHC Coordinates Are Relative to scaffold_42:14,700,000 from MonDom2 The size and complexity of the opossum MHC is similar to that of eutherian mammals, but the organization resembles that seen in non-mammals. The Class I and Class II regions are adjacent and somewhat interspersed. The antigen processing genes are closely linked to the Class I genes. The framework region does not contain Class I genes, as it does in eutherians. Like eutherians, the opossum MHC does not contain the third inducible proteasome subunit gene,*PSMB10.* Some genes present in the human MHC have not been found on the opossum scaffold_42*(MAS1L,* the histone cluster,*C6orf12, HCG2P4, RANP1, C6orf205, C6orf1, DHFRP, HCP5, HCG9P1, PPIP9, LST1, NCR3, AIF1, LY6G5B, LY6G6D, LY6G5E, C6orf10, RPL32P1, PPP1R2P1, HTATSF1P, MYL8P,* and*LYPLA2P1)*.

**Figure 2:**
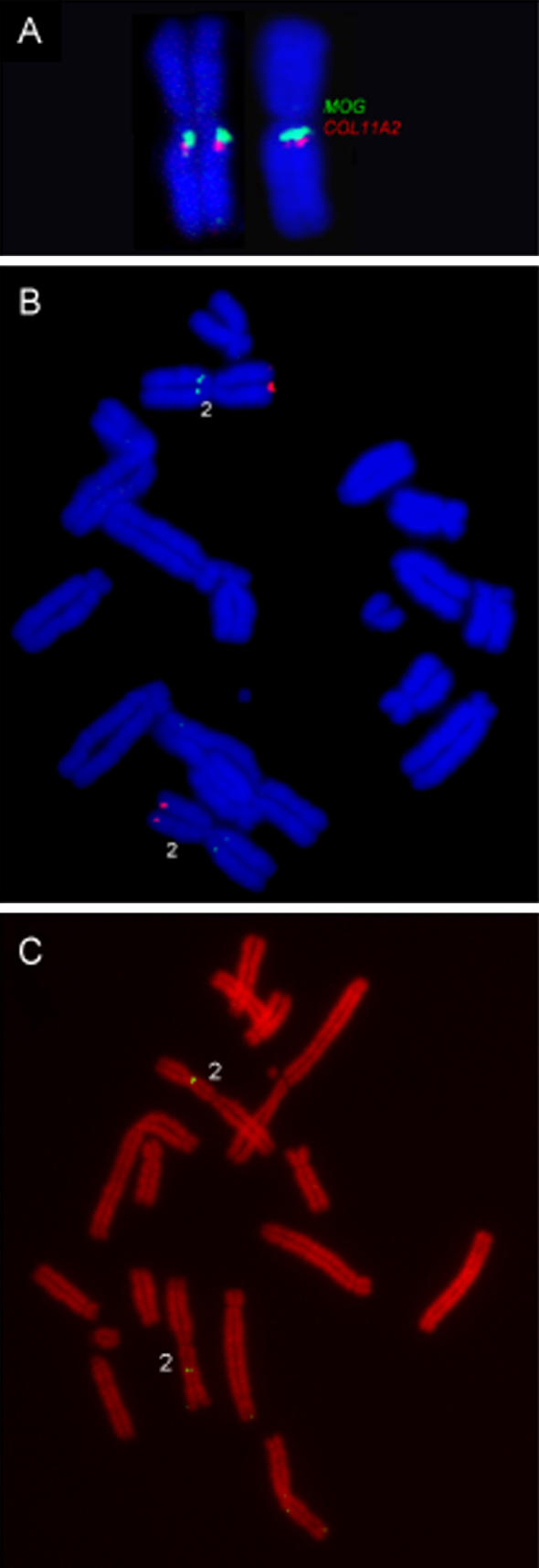
Fluorescence In Situ Hybridization of Opossum MHC-Containing BAC Clones on Opossum Metaphase Chromosomes (A) Co-localization of BACs containing*MOG* (162N8) (green) and*COL11A2* (27I16) (red) to show the orientation of the main MHC region at the centromeric region of 2q. (B) Co-localization of MHC-linked Class I*UG* (323O1) BAC to the centromeric region of 2q (green) and putative non-classical Class I*UB/UC* (253C16)–containing BAC to the telomeric region of 2p (red). (C) Localization of CD1 (969K11) to Chromosome 2p.

The opossum MHC is similar in size and gene content to the MHC of eutherian mammals. However, the organization of the opossum Class I, II, and III regions is different from that of eutherians and shows more similarity to the organization seen in birds and amphibians. Ostensibly, the main difference between the opossum MHC and that of eutherians is the position of the Class I genes (Figure 3). The opossum MHC has (1) a Class I/II region that contains interspersed Class I and II genes, (2) a “framework region” that is composed of only the framework genes in the opossum, but which also includes Class I genes in eutherians, (3) a Class III region with gene content and order highly conserved with eutherians, and (4) two extended regions that flank the MHC, corresponding to the eutherian extended Class I and II regions, and containing a very similar gene content and order.

**Figure 3:**
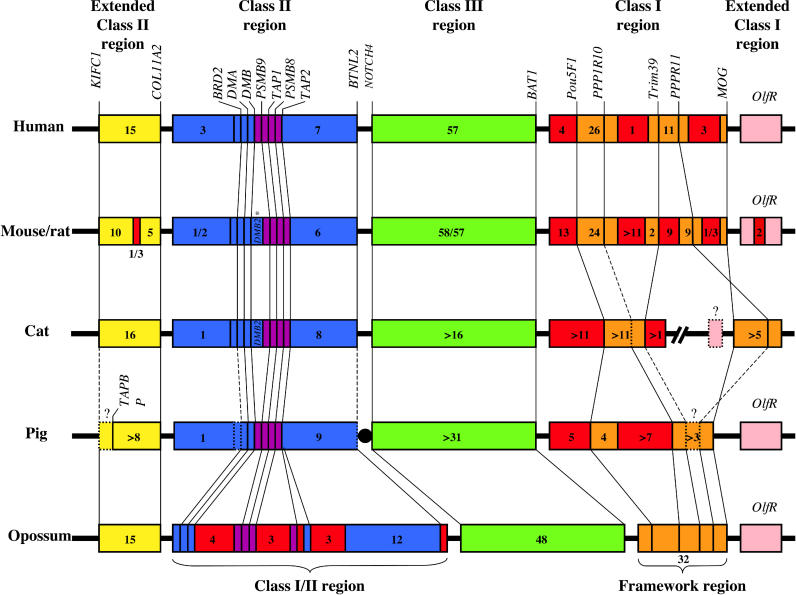
Comparative Map of the MHC Organization in Mammals The color code is the same as inFigure 1. Orthologous genes are indicated by connecting lines. Dashed lines and question marks represent unknown or uncertain data for a given gene or portion of the genome. The total number of genes is reported for each region or sub-region (pseudogenes are not included). Map is not drawn to scale. Unless specified, the small boxes represent single genes. This map was generated using data from [2,47]. The asterisk in the figure indicates that the duplication of DMB concerns only the mouse.

In the opossum, Class I and II regions are adjacent and interspersed rather than being separated by Class III (Figure 3). This unique arrangement of Class I and Class II loci has not been described in any other mammalian species. The proximity of Class I and II genes in opossum as well as non-mammalian vertebrates implies that Class I and II genes were originally located close together in the mammalian ancestor (Figure 4). This conclusion is further supported by the presence of Class I pseudogenes in the human Class II region, and the presence of both functional and non-functional Class I genes in the rodent Class II region (Figure 3) [1].

**Figure 4:**
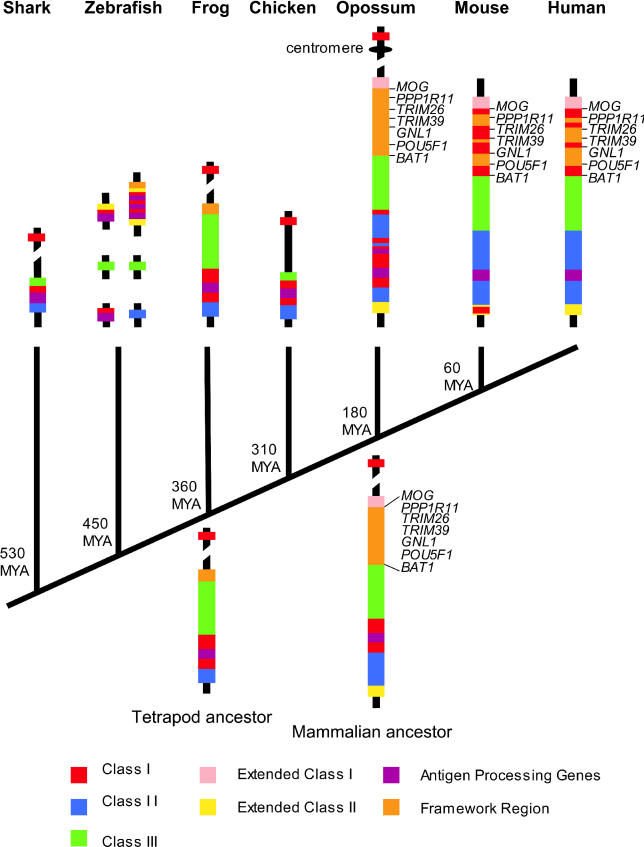
A Model of the Evolution of the Mammalian MHC Organization of the MHC in the mammalian ancestor is similar to that of non-mammals. Class I and II regions are adjacent and the antigen processing genes are found within Class I. Class Ib genes are located outside the MHC but on the same chromosome. The framework and extended regions have assembled. The framework gene order is conserved in both the opossum and eutherians. After the divergence of the eutherian lineage, Class I genes relocated to the framework region. MYA, million years ago.

In humans and mice, the MHC contains a region referred to as the Class I framework region due to the presence of a set of non-Class I or II genes, amongst which the Class I loci are interspersed [7]. The content and gene order of these framework genes are conserved between mice and humans. Remarkably, the opossum MHC contains a homologous cluster of framework genes (including*MOG, PPP1R11, TRIM26, TRIM39, GNL1, POU5F1,* and*BAT1*) next to the Class III region opposite the Class I/II region. These genes are in the same order as in the eutherian Class I framework region, but they lack the interspersed Class I loci (Figures 1 and4). This implies that a block of Class I framework genes was established near the MHC locus prior to the translocation of the Class I genes to this region in eutherians. Framework genes have not been reported in the MHC of non-mammals, but it is likely that the association of the framework region genes is ancient and that the framework region moved into the MHC en masse, given that five framework region genes appear on the same scaffold as Class III genes inXenopus tropicalis (Ensembl scaffold_547) (unpublished data).

InFigure 4, we present a model to explain how MHC organization evolved from a simple ancestral form to the complex forms seen today in therian mammals. We propose that in the MHC of a therian ancestor of marsupials and eutherians, Class I and Class II loci were located together at one end of the region, along with the antigen processing genes. A similar hypothesis was suggested previously based on studies of MHC organization in non-mammals [12]. Adjacent to the Class I and II regions was a gene-rich Class III region that already contained most of the genes present in human, mouse, and opossum MHC. The framework region, devoid of most or all Class I genes, assembled on the opposite side of Class III. The extended regions are present in the opossum as well as in eutherians, and therefore must have been present in the ancestral form. Studies have identified extended region genes in close proximity to MHC genes in teleost fish, despite the overall non-linkage of Class I, II, and III genes in these species [3,13,14].

The eutherian MHC Class I–III–II structure exemplified by rodents and primates evolved relatively recently. Class I genes must have relocated across the Class III region and interspersed between the framework genes after the divergence of marsupials and eutherians, but prior to the divergence of primates and rodents (∼60 million y ago) [15]. This process gave rise to the eutherian Class I region. It is unclear how or why the Class I genes relocated, but Class I loci appear to “migrate” in different species along with their frequent expansions and contractions. Specifically, Fukami-Kobayashi et al. [16] have suggested that long interspersed nuclear element (LINE) sequences can trigger genome fragment duplications, producing pairs of duplicated genome fragments. Perhaps, a series of duplicated genome fragments inserted themselves between framework genes in ancient eutherian mammals and have since been evolving via expansions and contractions in their new location.

The opossum MHC is unique in that Class I and II genes are interspersed and closely linked to antigen processing genes. The Class I expansion has occurred within the Class II region. The opossum MHC Class I/II region contains 11 putative Class I and ten Class II genes (predicted coding sequences available athttp://bioinf.wehi.edu.au/opossum). Class II loci include the non-classical*DMA* and*DMB* genes, whose homologs are found in birds and eutherians. Three marsupial-specific classical Class II gene families are present;*DA, DB* [17], and a newly discovered family that we have designated*DC* (Figures 1 and5).

**Figure 5:**
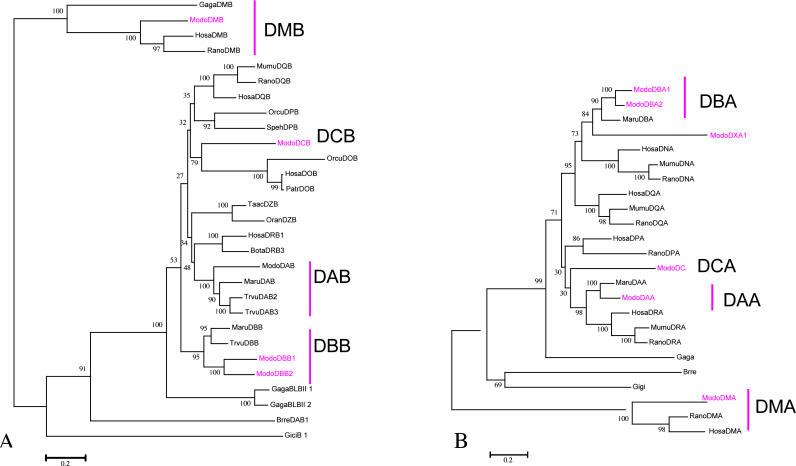
Phylogenetic Tree of the MHC Class II Genes (A) MHC Class IIB gene phylogeny based on full length amino acid sequences. Prior studies have named*DAB* and*DBB* [17]. Here we report a new Class IIB gene family,*DCB*. The*DMB* genes were used as the outgroup.*DAB* was not found in scaffold_42 and a cDNA sequence was used for the analysis. Physical mapping localizes*DAB* BACs to the centromeric region of 2q, and it appears this gene was not sequenced or was unable to be assembled. (B) MHC Class II A gene phylogeny. Location of IIA genes near IIB genes in scaffold_42 allowed designation of IIA genes to class IIB gene families: DA, DB, and DC. Bootstrap values are too low to be able to ascertain orthology with eutherian gene families.

Of the 11 Class I loci in the opossum MHC, only one;*UA1,* is known to have all the characteristics expected of a classical Class Ia locus by being both ubiquitously expressed and highly polymorphic.*UA1* transcripts have been detected in all tissues tested by RT-PCR and account for all previously described Class Ia cDNAs [18] (Figure 6A). The level of*UA1* polymorphism is also comparable to that of human HLA-A (N. Gouin, P. B. Samollow, M. L. Baker, and R. D. Miller, unpublished data). Expression of a single classical Class Ia gene in the opossum is unusual for a mammal, but not unprecedented in vertebrates. For example, both the chicken andXenopus laevis have a dominantly expressed single functional Class Ia molecule [19,20]. Unlike*X. laevis,* the opossum Class Ia gene,*UA1,* does not appear to have allelic lineages.

**Figure 6:**
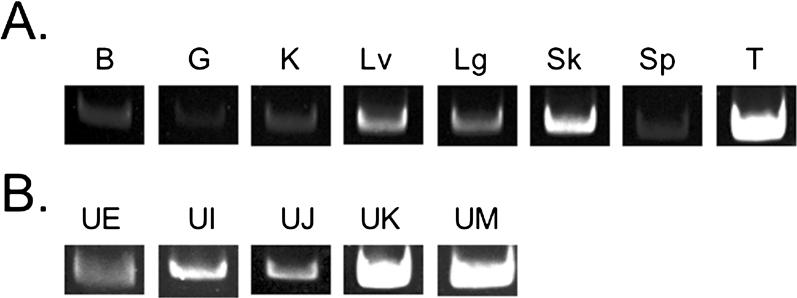
RT-PCR Results Demonstrating Class I Expression (A) RT-PCR results demonstrating Class I*UA1* expression in brain (B), gut (G), kidney (K), liver (Lv), lung (Lg), skin (Sk), spleen (Sp), and thymus (T). (B) RT-PCR results demonstrating expression of Class I*UE, UI, UJ, UK,* and*UM* in the thymus. Similar results were found for*UG* (not shown). Controls (water) were negative for all primer combinations (not shown).

Two of the Class I loci*(UA2* and*UH)* appear to be pseudogenes, because they lack a predicted ORF and have not been found expressed in any of the tissues examined (data not shown). Two other loci*(UF* and*UL)* have predicted ORFs, but their transcription has not been detected in any tissue so far and their functionality remains unknown. Five of the remaining Class I loci*(UE, UK, UJ, UI,* and*UM)* are all transcribed in the thymus (Figure 6B); however, each have tissue-specific expression, suggesting they are likely Class Ib in nature (S. D. Melman, M. L. Baker, and R. D. Miller, unpublished data).*UG* is transcribed in all tissues tested, including thymus, but the peptide binding sites are not polymorphic clearly suggesting it is a Class Ib gene (data not shown; N. Gouin, M. L. Baker, P. B. Samollow, and R. D. Miller,unpublished data). Overall, the majority of the 11 opossum Class I loci are transcribed. Since transcription is detected in the thymus, these have the potential to participate in T-cell selection, although other functions in thymic differentiation and T-cell development and regulation can not be ruled out.

The expressed Class I loci in the opossum Class I/II region are highly diverse, sharing as little as 49% nucleotide identity, and at most 83%, over exons 2, 3, and 4 among loci. A phylogenetic analysis of the Class I loci, including Class Ia and Ib loci from other species, is shown inFigure 7. Despite the sequence divergence of opossum Class I loci, they are phylogenetically related and probably evolved from common ancestral loci. This observation raises some questions about one of the current theories explaining the general absence of non-classical Class I genes within the MHC of non-mammals. It has been suggested that proximity of Class I genes to the antigen processing genes has constrained their divergence [12]. In eutherians, loss of this tight association by movement of the Class I genes away from the antigen processing genes may have resulted in increased plasticity that led to fluctuations in gene number and function and allowed Class Ib genes to reside in the MHC [12]. However, in the opossum, antigen processing genes have not constrained the diversification of the adjacent classical and non-classical Class I genes. It is unclear what selective advantage, if any, might have been gained by the separation of Class I from Class II or antigen processing genes in eutherians. Close linkage has been implicated in co-evolution of Class I genes and antigen processing genes [19,21–23]. Perhaps the Class I genes inM. domestica have evolved to be less constrained by their proximity to the antigen processing machinery, allowing them to duplicate and diversify in close linkage to the*TAP* and*PSMB* genes. Alternatively, co-evolution with the antigen processing machinery may have severely restricted Class I evolution in this marsupial, perhaps resulting in only a single locus performing the classical role.

**Figure 7:**
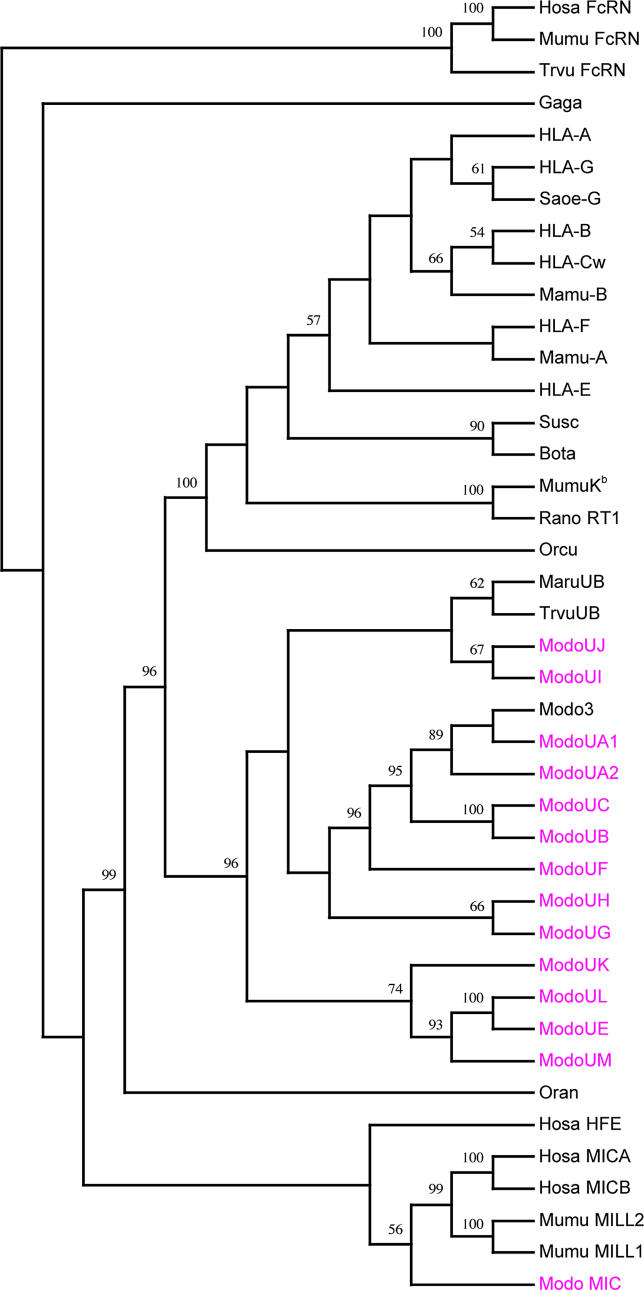
Phylogenetic Tree of the MHC Class I Genes MHC class I phylogeny based on nucleotide alignments corresponding to exons 2, 3, and 4 of MHC Class Ia and Ib loci fromM. domestica and representative vertebrate species.

Class I loci,*UB* and*UC,* were previously assumed to be linked to the MHC due to their high levels of sequence similarity to*UA1* [18], but surprisingly they are not found on the scaffold containing the MHC region (Figure 1) and have been localized to the telomere of Chromosome 2p, distant from the MHC at 2q centromere (Figure 2). Localization of Class I genes outside the MHC implies that these genes may have a non-classical role. In eutherians, the Class I loci lying outside the MHC are among the most divergent from Class Ia genes [2]. However, in non-mammals, genes closely related to Class Ia have been found outside the MHC, and in sharks, Class Ib genes found outside the MHC share very high levels of similarity to the Class Ia genes [8]. These non-mammalian genes have been designated Class Ib without elucidation of their functional roles, based on levels of expression and polymorphism. Currently, we do not have information about polymorphism levels of*UB* and*UC,* but their relatively low expression levels [24] may indicate evolution towards non-classical Class Ib functions.*UB* and*UC* are both flanked by marsupial-specific retroelements of the CORE-SINE type [24], which would be consistent with the role of such elements in Class I gene mobility [16] and may explain the recent relocation of*UB* and*UC* outside of the MHC [24]. The high level of sequence similarity of*UA, UB,* and*UC* raises the possibility that Class Ia genes can maintain their function when unlinked to the MHC.

Comparisons between MHC sequences of distantly related mammals highlight the conservation of the most important regulatory sequences, namely the SXY DNA motifs. Transcription of most MHC Class I and II genes is largely regulated by the Class II transactivator (CIITA), which interacts with several transcription factors, particularly those that bind to this motif [25,26]. Conservation of promoter elements in opossum Class I genes has been reported previously [24]. Using computational methods, we were able to identify SXY motifs upstream of most opossum MHC Class I and II genes. Eight SXY motifs were identified within 273 base pairs (bp) of the coding start in the opossum Class II genes (Table 1). Overall, these motifs were found to be conserved between eutherians and the opossum (Figure 8A). Eight SXY motifs were also identified upstream of opossum Class I genes (Table 1). We were not able to identify the SXY motif in genes*UK, UF,* and*UM*. Furthermore, the S motifs in the promoters of genes*UH, UI,* and*UL* appear to be weak with respect to the eutherian pattern. This suggests that the opossum Class I SXY regions have diverged from their corresponding eutherian motifs (particularly in the X motif;Figure 8B) more than the Class II SXY regions have. This is not unexpected given that Class II genes (classical and non-classical) are typically co-expressed whereas the non-classical Class I genes tend to evolve novel functions.

**Table 1:**
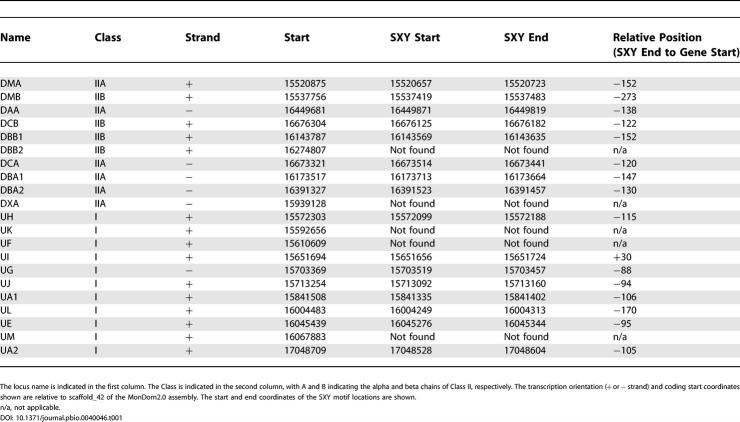
Coordinates of the Opossum Class I and Class II Loci Located within the MHC

**Figure 8:**
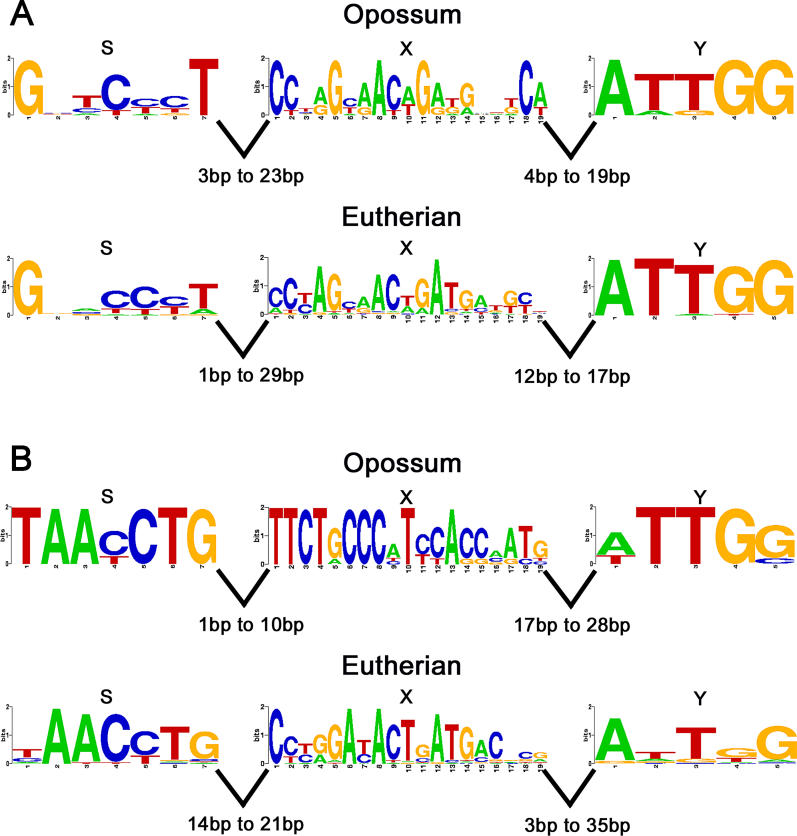
Analysis of the SXY Promoter Regions of MHC Class I and II Genes (A) SXY motifs in the MHC Class II genes. The LOGOs [48] of the corresponding position-specific scoring matrix models are presented. The height of each stack of symbols (*y*-axis) represents the information content in each position of the DNA sequence in log_2_ terms (bits of information) with a maximum value of 2. (B) SXY motifs in the MHC Class I genes.

Perhaps most significantly, our data also suggest an ancient relationship between the MHC and the natural killer complex (NKC), which contains C-type lectin natural killer (NK) cell receptor loci [27]. This relationship is drawn from the presence of two genes within the opossum MHC,*MIC* and*OSCAR*. Opossum*MIC* is the most distant homolog to the polymorphic human Class I genes*MICA* and*MICB* found to date (Figure 7). The*MIC* genes are Class I–related genes that encode ligands for NKG2D, a C-type lectin NK receptor [28].*MIC* genes are not found within the MHC of rodents. Instead, rodents have closely related genes, known as*MILL*. In a phylogenetic analysis, the opossum*MIC* is basal to a clade containing human*MICA/B* and the mouse*MILL1/2* genes (Figure 7). The function of rodent*MILL* genes is not yet known [29], but our results support a common evolutionary origin of*MIC* and*MILL* in eutherians. The presence of*MIC* in the opossum MHC, and its apparent absence in non-mammals, implies that*MIC*-like genes appeared before marsupials and eutherians diverged, and uniquely evolved into*MILL* in rodents.

The osteoclast-associated receptor*(OSCAR)* was first discovered as a receptor on mouse osteoclasts [30], but it has recently been shown to participate in antigen uptake and processing for Class II molecules in dendritic cells [31].*OSCAR* (also known as polymeric immunoglobulin receptor 3) is located within the leukocyte receptor complex (LRC) of humans, chimps, mice, and rats [32]. The presence of an*OSCAR* homolog within the opossum MHC is surprising. Using Genscan, we confirmed that the opossum*OSCAR* homolog contains an intact ORF and a predicted promoter. Human and opossum*OSCAR* share 47% identity at the amino acid level and are reciprocal best hits in BLAST searches (opossum*OSCAR* against human Refseq: best hit NP_573399.1 OSCAR isoform 4, e-value = 7e−66). Further, the presence of*OSCAR* in the opossum MHC suggests that involvement in antigen processing may be its original function.

MHC Class I molecules are ligands for NK cell receptors, so these two gene families must co-evolve. Keeping up with the rapid evolution of the MHC loci in response to pathogenic pressures is thought to have resulted in the independent evolution of two vertebrate NK receptor families, the C-type lectin and Ig superfamily types. In humans, the C-type lectin NK receptors are found on Chromosome 12 within the NKC [27]. The second NK receptor family contains the killer cell Ig-like receptors (KIR) and is encoded in the LRC on human Chromosome 19 [27]. The recent discovery of C-type lectin NK receptor genes in avian MHC [6] supports an ancestral association of the MHC and the C-type lectin genes of the eutherian NKC. Just as birds provide an ancestral link between the MHC and NKC [33], the two aforementioned opossum genes,*OSCAR* and*MIC,* provide links between the MHC and LRC.*OSCAR* is in the MHC in opossum (Figure 1) but in the LRC in humans and rodents.*MIC* genes are in the MHC of opossums and humans, whereas the related*MILL1/2* genes are in the LRC of rodents [34]. These observations support the existence of an ancestral genomic region in amniotes that probably contained MHC Class I loci and NK cell receptor genes of both the KIR and C-type lectin forms. This organization would have allowed both classes of NK receptors to co-evolve with their MHC ligands.

Recently,*CD1* genes were linked to the MHC of chickens, and may have been part of the primordial MHC [35,36]. Although a clear evolutionary relationship is evident between eutherian MHC Class I genes and*CD1, CD1* is not located within the eutherian MHC. The marsupial homolog of*CD1* has been identified in the opossum genome (M. L. Baker, S. D. Melman, and R. D. Miller, unpublished data). It is located on a separate scaffold (scaffold_13) from that containing the MHC and maps to Chromosome 2p (Figure 2).*CD1,* like the NK receptors, probably moved out of the MHC after the separation of mammals and birds but prior to the separation of eutherians and marsupials.

Comparative analyses of the MHC region in opossum and other species supports the idea that at one time in vertebrate evolution there was a single “immune supercomplex” of genes that contained MHC Class I and II, antigen processing genes*(TAP* and*PSMB), CD1,* and C-type and Ig-type NK receptor genes [37]. This complex is no longer found in any living species analyzed so far, but clues of its existence remain in extant genomes.

## Materials and Methods

### Sequence analysis and annotation

All results presented in this paper are based on the MHC-containing scaffold from the preliminary assembly of theMonodelphis domestica genome, MonDom2, released by the Broad Institute. The contig N50 length is 111 kb and the scaffold N50 length is 54 Mb (J. Chang, personal communication). MonDom2 is an interim assembly of unordered scaffolds. A finalM. domestica assembly with ordered scaffolds that are anchored to chromosomes is in preparation. Similarity features were identified by aligning all of the human proteins from the extended MHC that are represented in the RefSeq collection (Release 11,http://www.ncbi.nlm.nih.gov/RefSeq) against the opossum genome using TBLASTN [38] and extracting best hits. Known opossum MHC Class I and II genes were located by aligning their transcripts with the opossum genome assembly using BLASTN [38]. To exclude alignments with shared domains in other genes, a heuristic approach that identified the shortest chain of BLAST HSPs (highest-scoring segment pairs) having the best protein coverage was implemented. A single scaffold, scaffold_42, was found to contain most of the genes expected in the MHC. Known MHC transcripts from other marsupial species, including tammar wallaby*(Macropus eugenii)* and brushtail possum*(Trichosurus vulpecula)* were aligned with opossum scaffold_42 using TBLASTN and best hits were extracted. Gene predictions were made by running GENSCAN [39] on scaffold_42. To visualize these four feature annotation tracks, a MHC genome browser, based on GBROWSE [40], was set up. All features were clustered spatially (based on sequence position) using a custom PYTHON (http://www.python.org) script and these cluster features were hand curated. If a known opossum gene was present in a cluster, that gene replaced the cluster in the curated annotation. Class I cDNA sequences, obtained using 5′ and 3′ RACE, were aligned with scaffold_42 using BLAT [41]. Class III genes were annotated by extracting sequence from the cluster neighborhood and GENOMESCAN [42] was used together with the orthologous human protein. Class II and framework region genes were annotated using a combination of GENSCAN, alignment of orthologous proteins from multiple species with the predicted protein and hand curation of the putative gene. The annotation of the extended regions is based, in general, on similarity features only.

### BAC isolation and physical mapping

Overgos were designed for scaffolds with multiple BLAST hits to MHC genes in MonDom1.0 using the Overgo Maker program (http://genomeold.wustl.edu/tools/?overgo=1) and manually. High-density filters from the male opossum BAC library VMRC-6 (http://bacpac.chori.org/opposum6.htm) were probed with labeled overgos to identify MHC BAC clones. Overgo labeling and filter hybridizations were carried out using the BACPAC hybridization protocol (http://bacpac.chori.org/overgohyb.htm).

Overgos are as follows: 2447_ova, 5′-CAAAGGGAAGTGAGCAGAACCATG-3′ and 5′-CTGTATACATGGCTCTCATGGTTC-3′; 2447_ovb, 5′-ATGTGTTGTGCCTGAGGTTGTAGC-3′ and 5′-CACTTCTAGGCCCAATGCTACAAC-3′; 11936_ova, 5′-AAAGGGGAATTCTGGGGCATGAAG-3′ and 5′-CAGCGGTCCTCCTCTACTTCATGC-3′; 11936_ovb, 5′-CCAGGAGGACAGCATAAGTAGAAG-3′ and 5′-CTACCTAGGAGGTAGTCTTCTACT-3′; 14804_ova, 5′-CTTATCAGAGGCTAGCAGAGCTAA-3′ and 5′-CCTCCTCTGATTCTTTTTAGCTCT-3′; 14804_ovb, 5′-GTGCCCAAGGAACTTTCCAAATAC-3′ and 5′-CCCTGACACCTTCATAGTATTTGG-3′; 15208_ova, 5′-CCAGATAGGCTGATGAGCCTTTAC-3′ and 5′-TTGAAGACCATTGCATGTAAAGGC-3′; 15208_ovb, 5′-GGTCACCTCAAAGAGTACTGGGTT-3′, and 15208_3ovb, 5′-CCAAGTTAACTCATCTAACCCAGT-3′; 16657_5ova, 5′-ATAAGGAATCCTGGGCCTGAGGAT-3′ and 5′-TCATGGCTGCTCCTCTATCCTCAG-3′. The overgos used to isolate BAC 323O1 were: 5′-GGCTGAGGGATGGAGAGGAACAGC-3′ and 5′-AATTCGGTGTCCTGGAGCTGTTCC-3′. The overgos used to isolate BAC 253C16 were Mdo3OVF, 5′-CCTGCCGAGATCTCCCTGACGTGG-3′, and Mdo3OVR, 5′-CCTCGCCATCCCGCAGCCACGTCA-3′.

A BAC containing the*M. domestica CD1* locus was isolated from the opossum BAC library VMRC-18 (http://bacpac.chori.org/opposum6.htm). This clone (BAC 969K11) was identified because it contained BAC-end sequences that flank*CD1* in scaffold_13 in the currentM. domestica genome sequence assembly. Internal sequencing of BAC 969K11 was performed to confirm the presence of*CD1*.

PCR primers for each scaffold end were used to further screen putative positive BACs. Resulting products were cloned into pCR 4-TOPO cloning vector (Invitrogen, Carlsbad, California, United States) and sequenced using vector primers M13 forward and reverse. Primers: 2447_aF, 5′-AAAGGAGGGACTGTTGGAGTAAGC-3′, and 2447_aR, 5′-TCTTGGCTCTTCAGACACACTATCC-3′; 2447_bF, 5′-GTTGATGAATGTGTTGTGCCTGAG-3′, and 2447_bR, 5′-CCAGAACCCCTTTAGTGCCTATC-3′; 11936_aF, 5′-CCACATCCTATTCATCTTTGACCC-3′, and 11936_aR, 5′-GGCAATGCTGGTGACCTTCTAC-3′; 11936_bF, 5′-TGTGGGTTGGGTAGAGTGGAATC-3′, and 11936_bR, 5′-GCTTCTGCTGTTTTTATGGGCAC-3′; 14804_aF, 5′-TTGCCAGAGATTTCCCCAAAG-3′, and 14804_aR, 5′-CATTATGCCTAAACTGTGTGCCC-3′; 14804_bF, 5′-GGCTCAGAGAATGTAATGGGAGTG-3′, and 14804_bR, 5′-GCACAGGAACAGTTGAACAGTAAGC-3′; 15208_aF, 5′-CACTGCCAAACTTAGACTCTTCCC-3′, and 15208_aR, 5′-TGACCACCCAAAAGCCTTGAG-3′; 15208_bF, 5′-TATTCGGTCACCACACAGAGCC-3′, and 15208_bR, 5′-GCTTGCCATTCTCCAAAGGG-3′; 16657_aF, 5′-TTGGGTGCTTCAGTCAGAGAGTG-3′, and 16657_bR, 5′-TAGGAAAGAGGGATGCTGGGAG-3′.

MHC-positive BAC clones (85D1, 162N8, 27I16, 158D1, 169A24, 175J22, 207O10, 121D2, 78J18, 34E18, 53E6, 58H11, 323O1, 256G22, 249P7, 255G18, 258J24, 278M18, 32301, 256G22, 249P7, 255G18, and 278M18) and the CD1-positive clone (969K11) were differentially labeled and co-hybridized to metaphase chromosomes. BAC DNA (1 μg) was labeled by nick translation with either biotin-16-dUTP or digoxygenin-11-dUTP (Roche Diagnostics, Basel, Switzerland). Labeled probes were precipitated with 1 μg sheared opossum genomic DNA (size range between 200 and 700 bp) to suppress repetitive elements, and 50 μg salmon sperm DNA which acted as a carrier for the precipitation. Metaphase chromosomes from a maleM. domestica fibroblast cell line were prepared [43] and hybridized as described previously [44]. Fluorescence signals were captured on a Zeiss Axioplan epifluorescence microscope (Carl Zeiss, Thornwood, NewYork, United States) equipped with a CCD (charge-coupled device) camera (Spot RT; Diagnostic Instruments, Sterling Heights, Michigan, United States) and merged with DAPI images using IPlab imaging software (Scanalytics, Rockville, Maryland, United States).

### Class I expression analysis

Analysis of Class I gene expression in different tissues was done using reverse transcription PCR (RT-PCR). Total RNA was extracted using Trizol (Invitrogen) following manufacturers recommended protocols. Thymus, brain, and spleen RNA were extracted from tissues taken from a 9-wk-old maleM. domestica. All other tissues were from an adult (1-y-old) female. RNA was treated with TURBO DNA-*free* (Ambion, Austin, Texas, United States) to remove contaminating DNA. RT-PCR using total RNA samples from tissues shown inFigure 6 was performed using the GeneAmp RNA PCR Core Kit with oligo-dT priming following manufacturers recommended protocols (Applied Biosystems, Foster City, California, United States). The primers used for each Class I locus were designed to amplify exons 2 through 4, with the exception of*UA1,* which amplifies exons 2 to 3, and*UJ,* which amplifies exons 3 to 4. The primer sequences were:*UA1,* 5′-GCTCGGGGACTCGCAGTTCATCTCG-3′ and 5′-CCATCTGCAGGTACTTCTTCAGCCAC-3′;*UE,* 5′-CTGAACCGAGGTTCACAGCTGTA-3′ and 5′-GCTCACTTCCAGAGAGCATCTCC-3′;*UI,* 5′-AGAGTACTTCGACAGCCACAGCGCT-3′ and 5′-CCTCTTCCTGACCTGAAGTCAAAGA-3′;*UJ,* 5′-GCAACTTCAGGCGCGGGTTTAAAAG-3′ and 5′-CGGTACTGGTGATGGGTCACTCCTG-3′;*UM,* 5′-ATGCGAGTCAGAGCACCGAGATTGG-3′ and 5′-CTGAGTCAGAGGTGATATGGCGGGT 3′ and*UK,* 5′-GGGAGACCGCTCAGACTTTCGAA 3′ and 5′-CTTCATGGCTAATGTGATGAGTG-3′.

The size of the PCR products for*UA1, UE, UI, UJ, UK,* and*UM* are 487, 710, 666, 478, 393, and 267 bp respectively. The specificity of each RT-PCR amplification was confirmed by direct cloning (TOPO-TA cloning kit; Invitrogen) and sequencing (BigDye Terminator Cycle Sequencing Kit v3; Applied Biosystems) of the PCR products. Sequencing reactions were run on an ABI 3100 and chromatograms were analyzed using the Sequencher ver 4.5 program (Gene Codes, Ann Arbor, Michigan, United States).

### Phylogenetic analysis

Sequence alignments were made by first aligning amino acid translations to establish gaps corresponding to codon position. The MHC Class II trees were constructed using neighbor joining using pairwise deletion with Jones-Taylor-Thornton matrix (JTT) matrix model and 100 bootstrap replicates using MEGA 3. Species included are nurse shark (Gici), zebrafish (Brre), chicken (Gaga), echidna (Taac), platypus (Oran), rednecked wallaby (Maru), gray short-tailed opossum (Modo), brushtail possum (Trvu), rabbit (Orcu), cat (Feca), mouse (Mumu), mole rat (Naeh), human (Hosa). Class IIB Genbank references are as in [17,45]. Class IIA Genbank accession numbers are listed below in Accession Numbers.

The MHC Class I tree was constructed using Maximum Parsimony with 1,000 bootstrap replicates using the MEGA 3 program (http://www.megasoftware.net). The overall tree topology was reproduced using the Neighbor Joining and Minimal Evolution models.M. domestica Class I loci were named in the following manner:*UA1* was identified as the locus encoding the previously identified Class Ia transcripts (e.g., Modo3 included in this tree [18]), and*UA2* is a locus with high nucleotide identity (94% over exons 2, 3, and 4) to*UA1; UA1* and*UA2* are the only two Class I loci within the MHC similar enough to be considered two members of the same family.*UB* and*UC* were previously described [24] and named, and are not in the MHC (Figure 2).*UE* through*UM* are individual loci sharing 49% to 83% nucleotide identity over exons 2, 3, and 4 in a pairwise comparison. Species abbreviations are as inFigure 5 with the addition of rhesus macaque (Mamu), cottontop tamarin (Saoe), pig (Susc), cow (Bota), and rat (Rano).

### Analysis of SXY promoter regions

Known eutherian SXY motifs were used to build models to scan the promoters of the opossum genes. SXY motifs from 24 eutherian MHC Class II genes were collected and position-specific scoring matrix models [46] were constructed for each individual S, X, and Y motif. The distance between the individual motifs was also taken into consideration. The genes we used as training set were the human genes*HLA-DOA, HLA-DMA, HLA-DPA1, HLA-DQA1, HLA-DRA* (distal),*HLA-DRA, HLA-DOB, HLA-DMB, HLA-DQB1, HLA-DRB1,* and*HLA-DRB3;* the mouse genes*H2-DMa, H2-Ea, H2-Oa, H2-Ab1, H2-DMb1,* and*H2-Eb1;* and the rat genes*RT1-Da, RT1-DOa, RT1-DMa, RT1-Db1, RT1-Bb, RT1-DOb,* and*RT1-DMb*. These models were subsequently used to scan the promoters of the following ten opossum Class II genes:*DBA1, DBA2, DAA, DBB1, DBB2, DCA, DCB, DMA, DMB,* and*DXA1*. “Promoters” refers to 5 kb upstream and 1 kb downstream of the coding start.

Using the methodology described above, we also generated a model for the Class I SXY motif using data from 27 eutherian genes: the human genes*HLA-A, HLA-B, HLA-C, HLA-E, HLA-F,* and*HLA-G;* the mouse genes*H2-M10.1, H2-M10.2, H2-M10.3, H2-M10.4, H2-M10.5, H2-M10.6, H2-M2, H2-M3, H2-Q10, H2-Q7, H2-T10,* and*H2-T17*; and the rat genes*RT-CE10, RT1-CE11, RT1-CE13, RT1-CE15, RT1-CE16, RT1-CE3, RT1-CE4, RT1-CE5,* and*RT1-CE7*. A second model was constructed from two previously characterized*Monodelphis* MHC Class I–related genes:*UB* and*UC* [24]. The promoters of the 11*Monodelphis* genes (i.e.,*UH, UK, UF, UI, UG, UJ, UA1, UL, UE, UM,* and*UA2*) were scanned using these models.

## Supporting Information

### Accession Numbers

The GenBank (http://www.ncbi.nlm.nih.gov/Genbank) accession numbers for the genes and gene products discussed in this paper are scaffold_42 (CH465496); brushtail possum*FcRN* (AF191647) and*TrvuUB* (AF359509); chicken*Gaga* (AF013491) and (AY357253); cottontop tamarin*Saoe-G* (M63952); cow*Bota* (X80936); gray short-tailed opossum Modo-3 (AF125540),*ModoUB* (AF522352), and*ModoUC* (AF522352); human*DMA* (NP006111),*DNA* (M26039),*DPA* (M27487),*DQA* (M26041),*DRA* (M6033),*FcRN* (AF220542),*HFE* (AF115265),*HLA-A* (U03862),*HLA-B* (X91749),*HLA-Cw* (U06487),*HLA-E* (BC002578),*HLA-F* (BC009260),*HLA-G* (M32800),*MICA* (AY204547), and*MICB* (U95729); mouse*DNA* (M95514),*DQA* (M21931),*DRA* (U13648),*FcRN* (D37874),*K^b^* (U47328),*MILL1* (NM_153749), and*MILL2* (NM_153761); nurse shark*Gici* (M89950); pig*Susc* (AF014002); platypus*Oran* (AY112715); rabbit*Orcu* (K02441); rat*DMA* (NP942036),*DNA* (H004806),*DPA* (AH004805),*DQA* (X14879),*DRA* (Y00480), and*RT1* (X90376); rednecked wallaby*DAA* (previously*DRA*) (U18109),*DBA* (previously*DNA*) (U18110), and*MaruUB01* (L04952); rhesus macaque*Mamu-A* (AJ542571) and*Mamu-B* (AF157402); and zebrafish*Brre* (L19445).

## Abbreviations

BAC: bacterial artificial chromosome
bp: base pair
Class Ia: classical class I
Class Ib: non-classical class I
LRC: leukocyte receptor complex
MHC: major histocompatibility complex
NK: natural killer
NKC: natural killer complex
ORF: open reading frame
